# Annotations

**Published:** 1891-01-10

**Authors:** 


### Annotations.

**Mr. John Marshall, F.R.S.**

It is impossible to avoid thinking of the trite old saying, that "the world often knows nothing about its greatest men," as we read the simple announcement of the death of Mr. John Marshall, F.R.S. Mr. Marshall was a man of the most varied culture, of high personal character, and of peculiar authority on many questions that are of the greatest importance to the State. But he dies, and no part of the world even hears of his death, except those persons who scan the obituary columns of the daily newspapers. That, of course, is a fact of no importance to the dead, and probably of very little to the living who knew and loved him. We do not ask that a nation shall go into mourning for every eminent medical man who leaves the world; but we do feel that those whose life-work greatly serves the highest well-being of the state should at least be known of their contemporaries, and should be appreciated at their true worth. Medical men do not live for one another; they live for the world. If the admiration of a medical man's professional brethren were the only honour that he is entitled to expect, then Mr. John Marshall, both privately and officially, attained to an eminence which there was nothing beyond. By intellectual endowments and personal worth of the highest kind he rose from step to step until he became President of the General Medical Council of Great Britain. No man can ask a higher honour at the hands of the members of his own profession. But it is not just that a man who renders great services to the State shall be recognised only by a handful of persons, and those consisting exclusively of the initiated. Medicine, we repeat, is not for medical men, but for the world, exactly as religion, and law, and military services, and politics, and business are for the world. All those persons who rise to eminence in any of the callings just named are recognised as public benefactors. We claim for the distinguished among doctors that the same recognition shall be conceded to them. Whole columns might be filled with the record of the professional work done by Mr. Marshall, and the professional honours conferred one by one upon him. But of what avail would that be? The readers of The Hospital know these facts as well as we do, whilst the outside public would not spare time even to scan the page. We feel that Mr. Marshall, whilst raised to the highest eminence among his fellows, was not valued at his worth by the world at large. The members of the profession owe it to the dead to let those who are not members of it know that a great man has died from among them; and that the world at large owes to Mr. John Marshall a debt which it has never paid, and which now it never can pay.

**Sir Richard Quain.**

Dr. Quain has received the honour of a baronetcy. If he had received it twenty years ago the eternal fitness of things would have been better consulted. Many people will be glad, and probably not one will grudge him the honour. The medical profession owes something to Sir Richard Quain, and the public a good deal more. He has served the latter as a competent physician for fifty years; and that of itself is no small title to honour. But he has done more than that. He found the Royal College of Physicians, when he became a member of it, a close corporation, whose only function was to "hall-mark" metropolitan consulting physicians. He initiated a movement which resulted in converting the College into a central brain and pulsating heart for the vitalisation of the whole medical profession in this country. A work of that kind was almost as difficult as converting a stone Sphinx into living and breathing humanity. So long ago as 1860 Dr. Quain was an eminent person. He was then nominated by the Queen in Council as a member of the University of London. Twenty years ago he was made a Fellow of the Royal Society; and about the same time was appointed a Crown Representative of the General Medical Council of Great Britain. Sir Richard Quain's energies have by no means been confined within the limits of the medical profession. In 1865 a Royal Commission was created for the investigation of the cattle plague, which had for some time been decimating the herds of this country. Dr. Quain, with the Marquis of Salisbury, Earl Spencer, and others, constituted that important Commission. Sir Richard is the editor of the universally known *Quain's Dictionary of Medicine*, concerning which the highest praise that can be given to it is that it does not require a single word of commendation to any medical man in British practice. We have elsewhere discussed the general question of the public and State recognition of eminent medical men. That discussion was suggested by the tardiness with which an honour, long overdue, has been bestowed on Sir Richard Quain. We do not know where the fault lies, for we are not aware what political party Sir Richard has associated himself with. But that is a matter of no moment. Medicine has little to do with politics. What we feel is that merit of the highest kind, albeit unobtrusive, has been long overlooked, whilst vigorous self-assertion has, in innumerable instances, carried off honours to which it had no claim. We congratulate Sir Richard Quain on the public recognition of his merits, but we congratulate still more the members of the baronetcy whose ranks he has joined, and her Majesty's advisers who have at last atoned for the oversight of their predecessors. Sir Richard is an Irishman.

**Slaughtered Women and Children.**

The Hospital speaks in the name of philanthropy, and philanthropy is humanity. It has been said, and it is not yet contradicted, that the American troops at Pine Ridge, after overpowering the Indians by vastly superior numbers, slaughtered all the women and children they could come near. If the present writer were the despotic governor of America, he would command the instant return home of all those soldiers who took part in that affray, and have them, with their officers, shot dead, one by one. The American army has given to the Indians a striking object lesson; the American government ought now to give to the American army a striking object lesson. America is a country of white people: it has schools and universities; men of literature and men of science; poets and philosophers; it has tens of thousands of men who every Sunday stand up in the name of Jesus Christ to preach the peculiar doctrines of Christianity; and it has millions of men and women who profess to believe those doctrines, and who pay other men and women to carry them into various parts of the world, and to the territories of the Redskins, as well as elsewhere. America must have an army, and she cannot always prevent branches of her army from doing deeds that are not only condemned of God, but that have long been forbidden by men, even by fighting men. But is America just? Will she mete out justice to 500 men as she would to one man? Was there ever, anywhere, in the history of the world, a more cruel butchery than that of those Indian women and children? America began her history in a struggle for justice and freedom. In her youth she took the sword in her hand and carved out liberty with its good blade. In her prime she set free two millions of slaves. She has a noble record, and a fair fame; will she, of all civilised nations in the nineteenth Christian century, permit the latest pages of her history to be reddened with the life blood of women and children who had neither weapons nor men to defend them? If she does not punish, and punish to the last limits of justice, the unparalleled and unforgiveable murderers of those women and children, she will share their crime. Then every man with the conscience of a Christian, will thank his destiny that he was not born an American.

